# Exometabolomic-enabled discovery of compounds associated with *Escherichia coli* O157:H7 population dynamics in the lettuce phyllosphere

**DOI:** 10.1186/s12870-026-08917-9

**Published:** 2026-05-09

**Authors:** David F. Bridges, Cristian Jacob, Joseph E. Student, Rebecca B. Zhao, Ivan Simko, Maeli Melotto

**Affiliations:** 1 Department of Plant Sciences, University of California, Davis, CA USA; 2 Plant Biology Graduate Group, University of California, Davis, CA USA; 3 Departamento de Ciencias Vegetales, Facultad de Agronomía y Sistemas Naturales, Pontificia Universidad Católica de Chile, Santiago, Chile; 4 Horticulture and Agronomy Graduate Group, University of California, Davis, CA USA; 5 Sam Farr United States Crop Improvement and Protection Research Center, Agricultural Research Service, United States Department of Agriculture, Salinas, CA USA

**Keywords:** Fresh produce safety, Disease outbreak, *Lactuca sativa*, Human pathogens on leaves, Leaf chemistry

## Abstract

**Background:**

Contamination of fresh produce with human pathogens remains a serious public health and economic concern due to the absence of effective kill steps in the farm-to-fork chain. *Escherichia coli* O157:H7 has been implicated in multiple illness outbreaks linked to lettuce.

**Results:**

We examined the exometabolomic profile of 31 lettuce genotypes and identified variations in the chemical composition of both the leaf surface and the leaf apoplast, supporting variable bacterial growth. Furthermore, inoculation with *E. coli* O157:H7 induced changes in the overall chemistry of these leaf niches, allowing the identification of many niche-specific differentially accumulated metabolites (DAMs). Intersection analysis revealed little overlap of DAMs among the genotypes, suggesting that multiple metabolites, or a combination of metabolites, may contribute to bacterial persistence in phyllosphere niches. This information guided the design of metabolite cocktails to supplement bacterial inoculations of leaves. Overall, we observed that inhibitory and promoting cocktails significantly shifted the bacterial population titer to lower and higher, respectively, when compared to the control without metabolite supplementation. These shifts were more pronounced in some lettuce genotypes than others.

**Conclusions:**

These findings provide new insights into how the phyllosphere chemistry influences the survival of *E. coli* O157:H7, offering potential targets to mitigate food safety concerns through genetic and metabolic engineering.

**Supplementary Information:**

The online version contains supplementary material available at 10.1186/s12870-026-08917-9.

## Introduction

Lettuce (*Lactuca sativa* L.) is among the top 10 most valuable vegetable crops in the United States, with California and Arizona producing 80%-90% of all domestically consumed lettuce [1]. Unfortunately, leafy greens have been implicated in ~ 9% of all U.S. foodborne illness over the past three decades [2, 3]. Contaminated lettuce alone is estimated to account for nearly 60% of illnesses linked to leafy green consumption, with romaine responsible for 16–20% of *Escherichia coli* O157:H7 (hereafter O157:H7) cases across all food sources. Illnesses caused by Shiga toxin‑producing *E. coli* (STEC), particularly O157:H7, can result in severe disease manifestations such as hemorrhagic colitis and hemolytic uremic syndrome, which may lead to prolonged hospitalization or, in severe cases, death [4], highlighting the need for improved preventive measures.

Contamination of lettuce can occur at any point along the farm-to-fork continuum. However, risk models suggest that contamination by human pathogenic bacteria most often occurs in the production environment, particularly in open fields [3, 5]. Following a contamination event, pathogens like O157:H7 and *Salmonella enterica* can potentially survive epiphytically on the leaf surface, adhering to the phylloplane and aggregating in microhabitats such as trichomes or epidermal cell junctions [6–9]. The bacteria may also chemotactically migrate to internal tissues through wounds or natural openings such as stomata [10–12]. Once established in biofilms, surface crevices, or the apoplast, bacterial cells might be protected from sanitizers used during post-harvest washing [6, 8]. Consequently, current sanitation measures primarily aim to prevent cross-contamination during processing, as complete control of pathogens on contaminated leafy greens is not yet feasible [13, 14].

Bacterial survival in the phyllosphere depends on adaptation to chemically diverse microenvironments [15–17]. Metabolite profiles of both the phylloplane and apoplast are shaped by various factors, including plant genotype and age [18–20], plant-microbiota interactions [21–23], and abiotic stress [24, 25]. Nutrient availability on the leaf surface is a well-recognized driver of epiphytic colonization [26, 27]. For example, young lettuce leaves support higher populations of both O157:H7 and *S. enterica* than middle leaves, as young leaves exudates are enriched in nitrogen and carbon [28]. In contrast, certain secondary metabolites have been linked to reduced bacterial survival. In kale, flavonoid and phenolic levels varied with developmental stage and were inversely correlated with *Salmonella* populations in surface washes [24]. Likewise, in lettuce, cultivar-specific variation in flavonoid and anthocyanin content influenced *Salmonella* survival, with red oak-leaf tissues supporting lower populations than romaine [25]. Other traits, including cuticle composition [29] and surface structures like hydathodes, trichomes, and stomata, further contribute to phylloplane chemistry through exudate release [27]. Moreover, diurnal stomatal opening can release nutrients that attract *Salmonella*, promoting aggregation around stomata and potential internalization into the apoplast [10].

In the apoplast, human bacterial pathogens encounter distinct metabolomic landscapes across leafy green species [30]. In addition, pathogen presence can also trigger host immune responses that further reshape the leaf environment [31, 32]. Interestingly, lettuce cultivar-specific differences in immune responses and apoplastic metabolite modulation are associated with distinct internal survival of *Salmonella* and O157:H7 [32, 33]. Infiltration of these pathogens into tissues induces ROS and phenolic accumulation in lettuce leaves, with responses varying according to bacterial species and pre- versus post-harvest conditions [34]. Postharvest studies of lettuce cultivars selected for jasmonic acid-linked immunity revealed genotype-dependent differences in O157:H7 survival on cold-stored cut leaves, with higher pathogen inhibition linked to resistance to insects and phytopathogens and to elevated phenolic and anthocyanin contents [19]. Building on these findings, we characterized the metabolomic profiles of the leaf surface and apoplastic across multiple lettuce genotypes, associated these chemical landscapes to the survival of O157:H7, and validated these associations by causing shifts in bacterial survival in these niches with the addition of specific metabolites. Accordingly, this study was designed to link chemical features of distinct lettuce leaf niches with O157:H7 survival and to assess their biological relevance.

## Results

### O157:H7 shows variable net growth on the leaf surface and apoplast of the same leaf

**Fig. 1:**
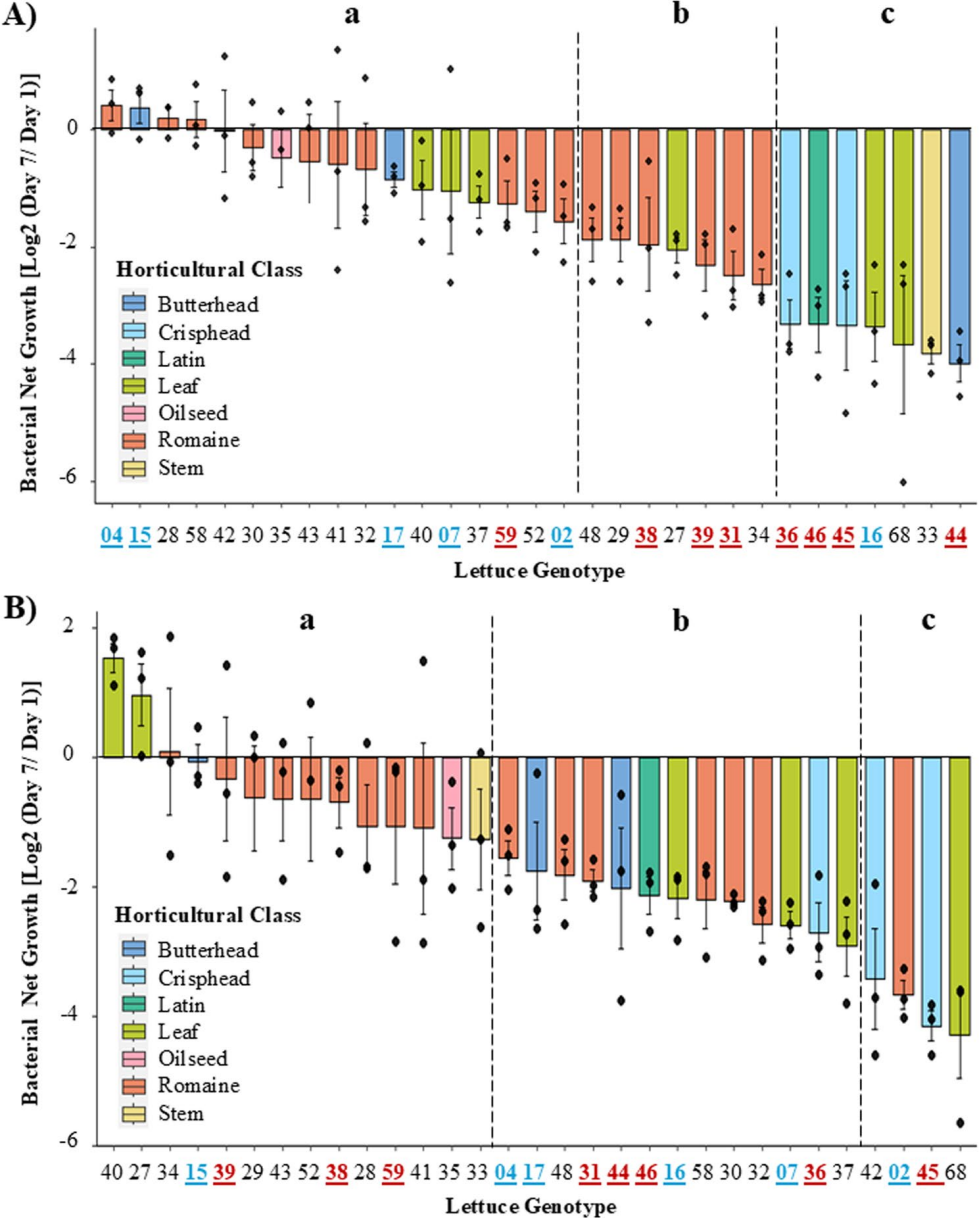
Net growth of *E. coli* O157:H7 recovered from the leaf surface (**A**) or from within the apoplast (**B**) of 31 lettuce genotypes, indicated by the codes on the X-axis and color coded by their horticultural type. Results are shown as means ± SE (*n* = 3) of Log_2_ ratio between bacterium population size at 7- over 1-day post inoculation (DPI). Statistically significant differences among the means were determined by ANOVA and the Scott-Knott post-hoc test (α = 0.05). Statistical groups are separated with vertical dashed lines and differing letters on the top. Bold, underlined genotype codes indicate the genotypes selected for metabolomic analysis, where red and blue letters indicated the genotypes used for metabolomic profiling at 1 and 7 DPI, respectively

To select lettuce genotypes with contrasting O157:H7 growth for metabolomic analysis, a panel of 31 genotypes representing seven horticultural types (butterhead, crisphead, Latin, leaf, oilseed, romaine, and stem) were screened for bacterial net growth on the leaf surface and apoplast. The net bacterial population change over seven days post inoculation (DPI) on the leaf surface of each genotype ranged from neutral to negative and the statistical analysis produced three groups of genotypes (Fig. 1A). While 16 out of the 31 genotypes are romaine, none of them were placed in the group that displayed the most significant reduction in O157:H7 growth. Furthermore, two butterhead genotypes (15 and 44) ranked at both extremes of the average O157:H7 net growth on the leaf surface (Fig. 1A).

To directly compare epiphytic and endophytic population dynamics, the net bacterial population change of O157:H7 in the leaf apoplast was estimated using the same leaves used for bacterial population size estimation on the leaf surface. Statistical analysis produced three groups of genotypes, with most positive and most negative average net growth in O157:H7 populations occurred in genotypes 40 and 68, respectively, which are leaf type (Fig. 1B).

Interestingly, the ordering and statistical grouping of the genotypes was not entirely consistent between the leaf surface and the apoplast. For instance, genotype 42 showed one of the largest and the lowest bacterial net growth on the leaf surface and the leaf apoplast, respectively. Only 12 out of the 31 genotypes were in the same statistical group for both leaf compartments (Fig. 1). Pearson correlation analysis between epiphytic and endophytic O157:H7 populations yielded a p-value of 0.25 (Fig. S1), supporting notion that the chemical composition of these two compartments may be distinct, contributing to differential survival of O157:H7 on and in the same leaf.

### Leaf exudate from different lettuces supports variable O157:H7 growth

**Fig. 2:**
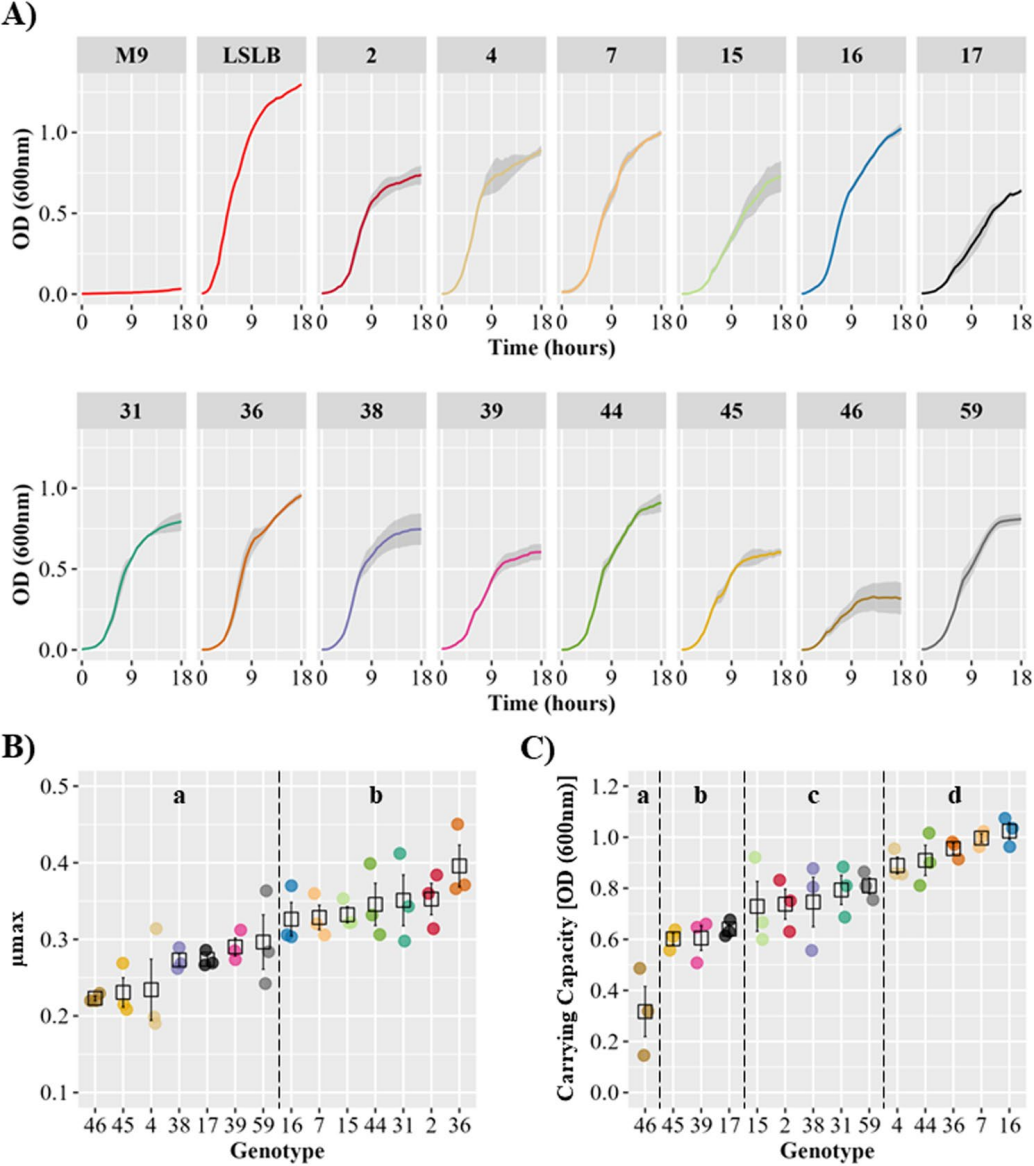
Growth of O157:H7 in different media, including rich LSLB, minimum M9, or apoplastic wash fluid (AWF) extracted from different genotypes. **A** Bacterial growth curves are shown as the mean 600 nm optical density (OD) ± standard error (*n* = 3), indicated by shaded area outlining the curves, up to 18 h of culturing. Codes on top of the plots refer to the genotype used for AWF extractions. **B**,** C** The graphs show the maximum growth rate (µmax) (B) and the maximum population density (carrying capacity) (C) of the growth curves. These parameters were estimated using the growthrates package in R. Significant differences among group means (*n* = 3) were assessed via ANOVA followed by a Scott-Knott post-hoc test (α = 0.05)

To assess whether the pre-existing leaf metabolome profile could cause differences in bacterial growth among genotypes, growth curves of O157:H7 in apoplast wash fluid (AWF) were generated. Because OD₆₀₀ measurements in AWF may be influenced by its composition, these assays were interpreted as relative indicators of bacterial growth trends rather than absolute measures of bacterial abundance. Fourteen lettuce genotypes were selected for this assay based on the O157:H7 enumeration data, representing the phenotypic variability of O157:H7 population net growth and multiple horticultural types (Fig. 1). Using the AWF as a growth medium, we observed that O157:H7 grows by solely utilizing water-soluble chemicals extracted from leaves of all genotypes and in intermediate levels between the rich medium LSLB and minimum medium M9 (Fig. 2A). However, the maximum growth rate (µmax) and the maximum bacterial number (carrying capacity) varied significantly (*p* < 0.0001) among AWF cultures (Fig. 2BC). The µmax ranged from 0.229 ± 0.003 (genotype 46) to 0.396 ± 0.022 (genotype 36) and the statistical analysis separated the genotypes into two groups (Fig. 2B). Likewise, the carrying capacity in the AWF medium, determined as the maximum OD_600_, spanned from 0.32 ± 0.10 (genotype 46) to 1.02 ± 0.03 (genotype 16) and statistical analysis separated the genotypes into four groups (Fig. 2C). Notably, fast growth rates did not always correspond to the largest carrying capacity among the genotypes when the bacterial population entered the stationary phase early as illustrated in Fig. 2A.

To assess whether O157:H7 growth in vitro (Fig. 2) correlated with population sizes *in planta* (Fig. 1B), we performed Pearson’s correlation analyses between these datasets. Bacterial population sizes at 1 or 7 DPI inside leaves showed no significant correlation with µmax (*r* = 0.29 − 0.18, *p* = 0.31–0.53), whereas they showed moderate correlations with culture carrying capacity (*r* = 0.36–0.45, *p* = 0.21 − 0.11) (Fig. S2). These results indicate that the lettuce pre-existing metabolome may provide varying sources of nutrients to support distinct O157:H7 growth. In addition, the combination of constitutively produced and induced metabolites in response to the bacterium are relevant for its population dynamic in leaves.

### Exometabolomic profile shifts in response to O157:H7

To take a step closer to identifying specific or a combination of metabolites associated with O157:H7 growth or decline on the leaf surface or in the apoplast, we conducted an untargeted exometabolomic analysis of leaf surface wash fluid (SWF) and AWF of 14 selected genotypes plants inoculated with either O157:H7 or a mock solution. Furthermore, recognizing that lettuce immune responses to O157:H7 occurs within 24 h of leaf contamination [35, 36], and metabolic susceptibility to O157:H7 colonization occurs later in lettuce interactions with this bacterium [32], two sets of lettuce genotypes were chosen for metabolomic analysis at 1 DPI or 7 DPI. As such, genotypes spanning the spectrum of bacterium net growth representing five horticultural types (Fig. 1) were chosen as follows: 31, 36, 38, 39, 44, 45, 46, and 59 for metabolomic profiling at 1 DPI, and genotypes 02, 04, 07, 15, 16, and 17 for metabolomic profiling at 7 DPI. For exometabolomic profiling, the SWF and AWF were collected from the same leaf sample so their chemistries could be compared to some extent (Fig. S3). Principal component analysis (PCA) of all biological replicates from all genotypes (Datasets S1-4) showed that mock- and O157:H7-inoculated samples had some overlap for some genotypes or grouped separately for others (Fig. S4), suggesting that O157:H7 may cause variable metabolic changes in different genotypes.

To evaluate the impact of O157:H7 on the leaf metabolomic profile, we performed hierarchical clustering of all detected metabolites, grouped by detection platform, days post‑inoculation (DPI), and leaf compartment (SWF or AWF) (Fig. S5). Overall, clustering patterns indicated that both lettuce genotype and inoculation status influenced exometabolomic profiles. Across metabolite classes and sampling times, some genotypes exhibited clear shifts in clustering following O157:H7 inoculation, with mock‑ and bacterium-inoculated samples separating into distinct groups, whereas other genotypes showed little to no separation between treatments. These effects were observed for both primary metabolites and polyphenols/flavonoids and differed between leaf compartments and DPIs, indicating context‑dependent metabolomic responses. In contrast, several genotypes displayed more attenuated responses, with mock and inoculated samples clustering closely together across conditions, suggesting limited exometabolomic changes following O157:H7 exposure. Collectively, these results indicate that O157:H7 elicits genotype‑dependent and compartment‑specific metabolomic responses in the lettuce phyllosphere during colonization.

Although the exometabolomic analysis allowed the identification of 4,244 and 4,329 metabolites at 1 and 7 DPI, respectively, only 515 and 421 of them are functionally annotated (Datasets S1-4), and subsequent analyses were focused on this subset of annotated metabolites.

### Identification of metabolites associated with O157:H7 presence in the phyllosphere

**Fig. 3:**
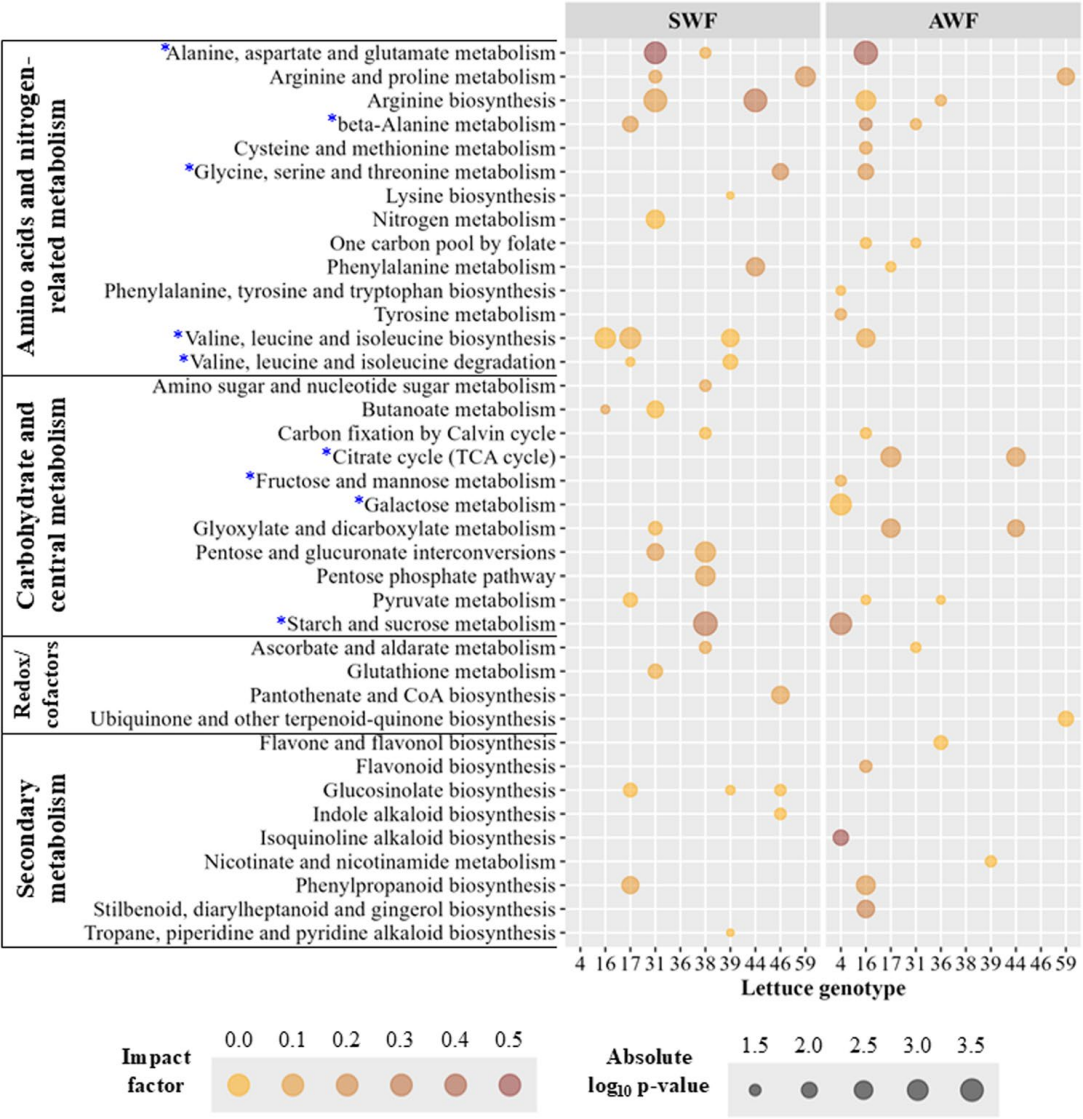
Pathway enrichment analysis of metabolites significantly accumulated (bacterium vs. mock) in the surface wash fluid (SWF) and apoplast wash fluid (AWF) of the lettuce genotypes. Significantly enriched pathways (*p* < 0.05) were identified using KEGG-based pathway analysis in MetaboAnalyst6.0 via the hypergeometric test (Dataset S7). Bubble size corresponds to the absolute Log_10_ p-value and color intensity represents the pathway impact score, which reflects the topological importance of mapped metabolites within each pathway. Pathways marked with a blue asterisk are associated with compounds selected for the inoculation cocktails

To identify metabolites that can potentially change the O157:H7 population size on the leaf surface and in the leaf apoplast, we identified differentially accumulated metabolites (DAMs) in each genotype. To this end, we calculated the relative accumulation of metabolites in the O157:H7- versus mock-inoculated samples as Log_2_ FC and set the threshold for significance at values ≤ -1 or ≥ 1 and *p* < 0.05. This analysis allowed for the identification of 339 DAMs at 1 DPI (Dataset S5) and 287 DAMs at 7 DPI (Dataset S6). The number of annotated DAMs varied among the genotypes, ranging from 4 in genotype 02 to 137 in genotype 16 (Table 1). Interestingly, the metabolism of amino acids, sugars, and flavonoids were significantly enriched (*p* < 0.05) among genotype-specific DAMs (Fig. 3, Dataset S7).

**Table 1:** Number of differentially accumulated metabolites (DAMs) detected in the leaf surface wash fluid (SWF) or the apoplast wash fluid (AWF) collected from each genotype at one- or seven- days post inoculation (DPI) and within the primary metabolites (PM) or polyphenols and flavonoids (PF) platforms. Statistical analysis to identify significant DAMs are included in Datasets S5 and S6

		# DAMs – SWF		# DAMs – AWF	
	Genotype Code	Total	Annotated	Total	Annotated
1 DPI	31	269	36	395	16
	36	330	31	218	17
	38	259	52	507	36
	39	266	25	384	32
	44	325	26	215	31
	45	236	19	199	22
	46	222	19	163	11
	59	271	24	171	26
7 DPI	02	132	4	154	18
	04	200	7	260	25
	07	201	16	468	35
	15	117	15	250	10
	16	149	13	1211	137
	17	268	44	369	21

The large number of DAMs prompted us to set some criteria for the selection of metabolites to be validated *in planta* as follows: (a) the accumulation of the metabolite should be significantly (*p* < 0.05) different in O157:H7-treated samples in comparison to the mock treatment in at least one genotype; (b) metabolites should be commercially available with minimum safety risks for manipulation; (c) metabolites should not cause any visual damage to the leaves; and (d) indication that the metabolites had some effect on bacterial growth based on previous functional annotations. Furthermore, an intersection analysis showed that some metabolites were identified as DAMs across more than one genotype (Fig. S6, Dataset S8), supporting their selection for further testing.

To demonstrate the impact of specific combinations of metabolites on O157:H7 colonization of the lettuce phyllosphere, we designed chemical cocktails predicted to be promoting or inhibitory of bacterial growth in the phyllosphere. As the bacterial net growth on the leaf surface (Fig. 1A) and the leaf apoplast (Fig. 1B) of each lettuce genotype was not always at the same level and the metabolomic profiles of these leaf locations differed (Fig. S5), location-specific cocktails were created (Table 2) following the criteria described above. Each cocktail contained five metabolites at 0.01 M for each of them.

**Table 2:** Chemical composition of cocktails to validate metabolomic predictions. Each cocktail contained 0.01 M of the indicated chemical

Location	Cocktail	Chemical
Leaf Surface	Promoting	D-(+)-Glucose
		Sucrose
		D-(-)-Quinic Acid
		4-Hydroxyphenylacetic acid
		L-(+)-Asparagine
	Inhibitory	Myo-inositol
		D-Sorbitol
		Fumaric Acid
		Citric Acid
		L-Valine
Leaf Apoplast	Promoting	D-(+)-Glucose
		D-(+)-Maltose
		Succinic Acid
		α-Ketoglutaric acid
		Glycine
	Inhibitory	D-(+)-Trehalose
		Glycerol
		Benzoic Acid
		Lactic Acid
		L-Alanine

To gain insight into the relative accumulation of selected DAMs and their derivatives identified with the exometabolomic analyses across all genotypes, heatmaps were created (Fig. S7). Interestingly, some metabolites showed distinct accumulation patterns in genotypes that support increased or decreased O157:H7 populations. As an example, quinic acid and its derivatives accumulated at relatively highest levels on the leaf surface of genotypes 16 and 44 (Fig. S7A) where the bacterial population declined the most, whereas the level of these metabolites was lowest on genotypes 04 and 15 where the bacterial population increased (Fig. 1A). Furthermore, the levels of glycine and its derivatives were lowest in the apoplast of genotype 45 (Fig. S7B) where bacterial populations were also the lowest (Fig. 1B), whereas these metabolites accumulated at relatively higher levels in genotypes 38, 39, and 59 where the bacterial populations were also the highest.

### Metabolic supplementations alter O157:H7 growth in the lettuce phyllosphere

**Fig. 4:**
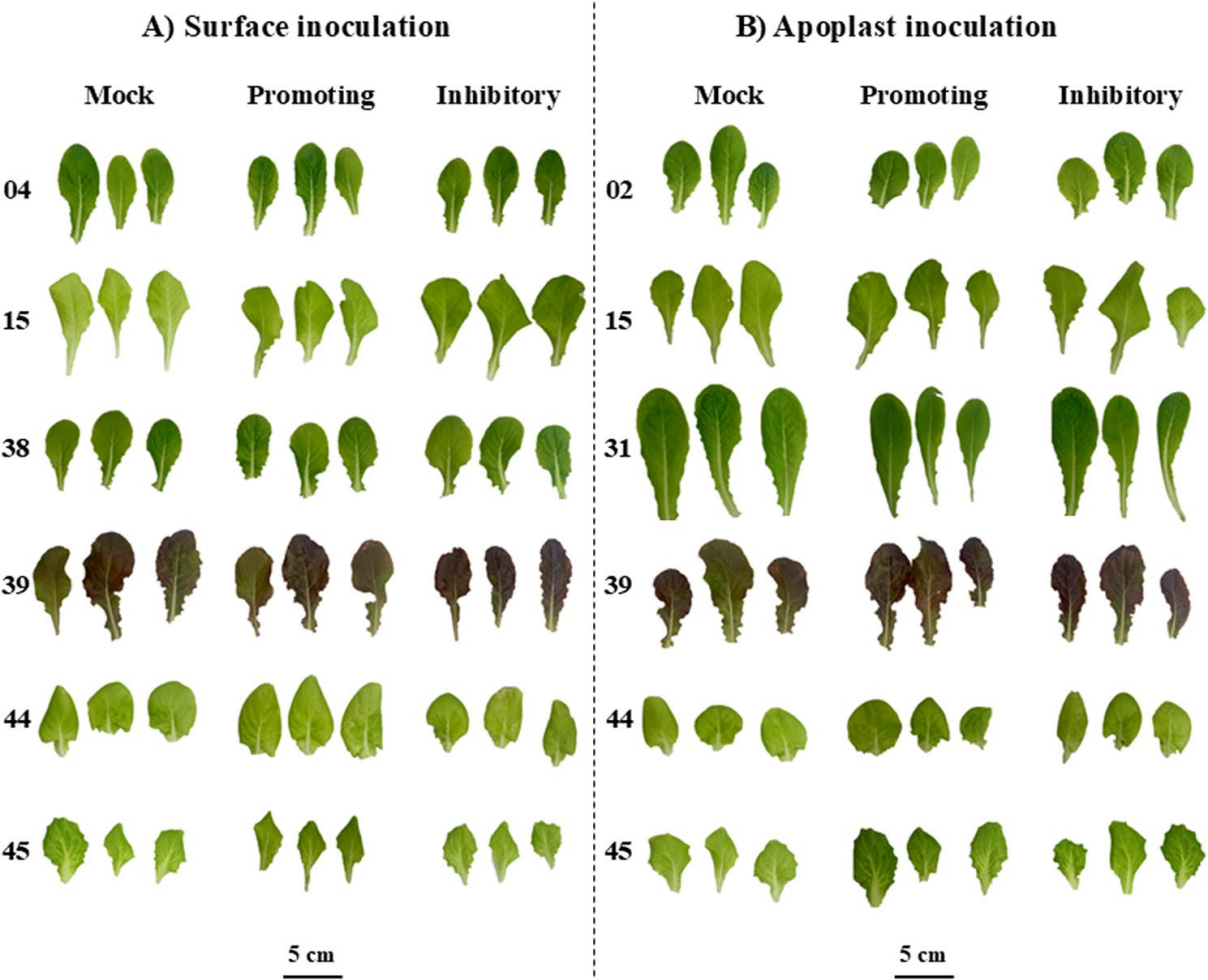
Representative images of leaves inoculated with the indicated cocktail (mock, promoting, or inhibitory) at seven days post-inoculation. **A** Surface inoculation. **B** Apoplast inoculation. Numbers on the left correspond to the lettuce genotype codes

Six lettuce genotypes were selected for inoculation with O157:H7 supplemented with each chemical cocktail or water control. These genotypes represent two horticultural types (romaine and butterhead) that are at the opposite ends of O157:H7 net growth spectrum, and a crisphead genotype that showed one of the lowest bacterial net growths in both compartments of the phyllosphere (Fig. 1). We reasoned that inoculum supplementation with a promoting or inhibitory cocktail would shift the bacterial population size to a higher or lower level, respectively, as compared to the non-supplement control. Leaves treated with metabolite cocktails in the absence of O157:H7 exhibited no visible symptoms or signs of tissue damage (Fig. 4).

**Fig. 5:**
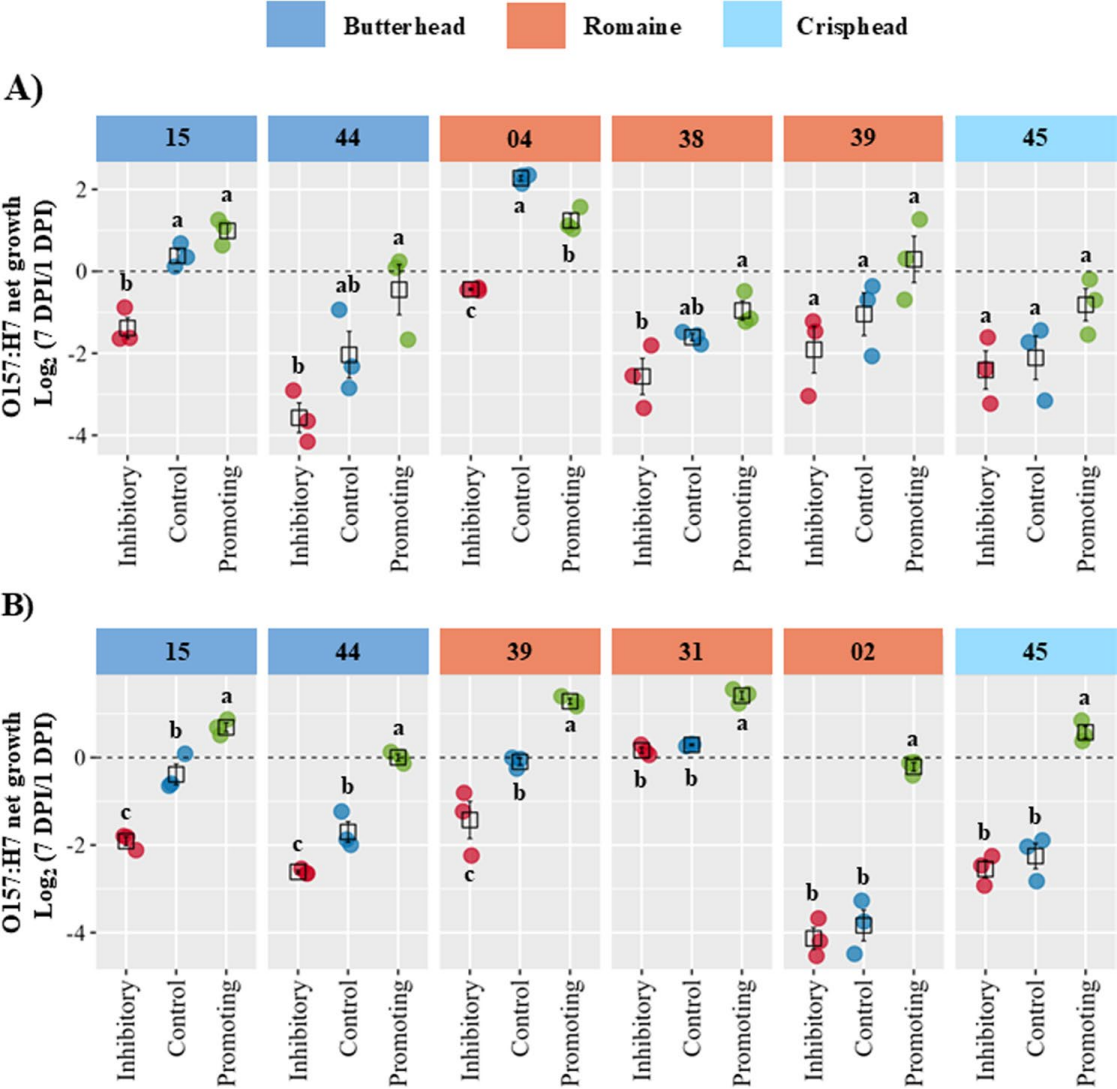
Metabolic supplementation alters O157:H7 net growth in the phyllosphere. **A**,** B** O157:H7 net growth on the leaf surface (**A**) or within the apoplast (**B**) of various lettuce genotypes (codes are on the top of the graphs) after inoculation with 1 × 10^6^ CFU/mL O157:H7 supplemented inhibitory, control, or promoting metabolic solutions (Table 2). Results are shown as means ± standard error (*n* = 3) of Log_2_ (7 DPI / 1 DPI). Empty squares represent the mean of three data points (colored circles). Statistical differences among the means were assessed through ANOVA and Tukey´s test (α = 0.05) represented with differing letters on top of the squares. DPI = days post inoculation

Leaf surface inoculation of genotypes 04, 15, 38, 39, 44, and 45 with O157:H7 supplemented with the inhibitory cocktail resulted in the least mean net growth for all genotypes. However, for genotypes 39 and 45 the difference was not statistically significant (α = 0.05) compared to that of on leaf surface supplemented with control solution (Fig. 5A). Similarly, the O157:H7 inoculum supplemented with the promoting cocktail resulted in the highest mean net growth for all genotypes, except genotype 4. The net growth of O157:H7 supplemented with the promoting chemical cocktail was statistically similar to that of on leaves without chemicals (control samples). However, O157:H7 with a promoting cocktail still grew significantly higher compared to the inhibitory cocktail treatment on the leaf surface of genotypes 04, 15 ,38, and 44 (Fig. 5A).

In the apoplast, supplementation with the inhibitory cocktail significantly decreased O157:H7 populations in all genotypes, namely 02, 15, 31, 39, 44, and 45, compared to supplementation with promoting cocktail (Fig. 5B). No supplementation resulted in an intermediate O157:H7 net growth in genotypes 15, 39, 44. Comparatively, the promoting cocktail treatment resulted in O157:H7 net population growth significantly (α = 0.05) higher in all six genotypes tested (Fig. 5B). Cocktails did not induce any visual damage to the inoculated.

While the exact underlying mechanisms are yet to be elucidated, these results demonstrate that the level of specific metabolites available in the extracellular spaces of the phyllosphere can significantly alter O157:H7 population growth dynamics *in planta*.

## Discussion

Understanding molecular, chemical, and morphological traits that influence the differential survival of human pathogenic bacteria in the phyllosphere is critical for integrating plant-based approaches into food safety strategies [19, 37, 38]. In this study, we identified lettuce genotypes exhibiting markedly contrasting net growth of O157:H7 as epiphytes and endophytes over the experimental period, (Fig. 1). Several genotypes supported positive or neutral bacterial net growth, indicating a greater capacity to sustain O157:H7 populations and potentially posing a higher food safety risk. Dissecting the metabolism associated with pathogen growth might be useful to breed out of the lettuce germplasm [39]. Conversely, some other genotypes showed significant reductions in bacterial populations, offering an opportunity to investigate plant traits associated with pathogen suppression [40].

Lettuce horticultural classification did not necessarily predict O157:H7 colonization dynamics. For instance, genotypes 15 and 44 (butterhead) and genotypes 02 and 39 (romaine) exhibited divergent bacterial net growth patterns on the leaf surface and in the apoplast, respectively (Fig. 1). These results support the notion that cultivars within the same horticultural type can differ substantially in traits influencing microbial interactions [19, 32]. Furthermore, we observed distinct net bacterial growth between the leaf surface and apoplast within the same genotype (Figs. 1 and S3), indicating that colonization dynamics are niche-specific on the same leaf as it has been observed in other pathosystems such as leaf-*Botrytis cinerea* interactions [41]. These findings prompted further investigation into the pre-existing and induced chemistry of these phyllosphere niches. Although the surfactant Silwet L‑77 used for inoculations may influence leaf surface properties, its consistent application in both mock- and bacterium-inoculated samples supports comparative interpretation of treatment-dependent effects. Resident phyllosphere microbiota can further modulate chemical profiles during plant–microbe interactions; therefore, future studies assessing native microbial communities will be necessary to extend these findings to more ecologically complex systems. Finally, because plants were grown under both controlled and field conditions, environmental factors may have contributed to variation in metabolite profiles and bacterial dynamics, an effect that was considered when interpreting genotype‑ and treatment‑dependent patterns.

The lettuce phylloplane and apoplast, where bacteria reside and form biofilms [42], are distinct chemical environments (Figs. S3 and S4). Corroborating previous findings [32, 43–45], we observed that the overall leaf surface and apoplast metabolite landscape varies substantially across lettuce types and cultivars, including differences in sugars, amino acids, pigments, and vitamins (Datasets S1-4), which may influence organoleptic, nutritional, and bioactive properties of the phyllosphere. O157:H7 triggers lettuce immune responses, such as ROS burst and callose deposition [35, 46, 47], which may also reshape the chemical environment. As with most untargeted metabolomics approaches, only a subset of detected features could be confidently annotated, which limits biological interpretation at the level of individual metabolites despite the use of comprehensive reference libraries [48]. Importantly, our metabolomic data suggest that these lettuce-O157:H7 interactions are also accompanied by broader, genotype-dependent shifts in the chemical composition in both leaf compartments (Figs. S5 and S6; Table 1), which go beyond plant immune responses. Accordingly, differences between results obtained with AWF assays and pathogen persistence within intact tissues are expected (Fig. S2), as AWF captures a static metabolite availability, whereas bacterial dynamics *in planta* may reflect additional spatial, diffusion‑related, and host‑associated constraints. To explore this possibility, we leveraged our metabolome data to design five-compound cocktails predicted to have either promoting or inhibitory effects on pathogen growth on/in leaves (Fig. S7; Table 2).

Each cocktail contained DAMs representing carbohydrates, amino acids, and organic acids to maintain a balanced chemistry and pH of the extracellular environment and avoid leaf cell damage (Fig. 4). As such, the inhibitory cocktail supplementing surface inoculations included: (i) myo-inositol, a sugar alcohol nonfermentable by O157:H7 [49], which is known to mediate root colonization by beneficial bacteria [50, 51] and participate in plant stress responses and regulatory networks [52, 53]; (ii) sorbitol, a sugar alcohol that most O157:H7 strains cannot ferment [54, 55], which can enhance plant defenses against generalist pathogens such as *Alternaria alternata* [56, 57] and stimulate suberin and lignin deposition in wounded tissues [58]; iii-iv) fumaric and citric acids that have antimicrobial properties against foodborne pathogens in diverse matrices, including in vitro systems [59], tabbouleh salad [60], and apple juice [61]. These organic acids can also inhibit *E. coli* biofilm formation [62, 63]; v) valine, an amino acid capable of inhibiting *E. coli* K12 growth through the induction of isoleucine starvation [64–66].

The promoting cocktail used to supplement leaf surface inoculations contained: (i) glucose, a central component of O157:H7 carbon metabolism [67, 68], which can enhance O157:H7 retention on spinach leaves [8]. However, glucose concentration alone might not be strictly associated with differential persistence across lettuce types [20] or improve bacterial survival on lettuce leaves [28]; (ii) sucrose, a sugar that is poorly metabolized by most *E. coli* strains [69], but it can enhance O157:H7 survival under stress conditions [70, 71]; (iii) 4-hydroxyphenylacetic acid, an organic acid with auxin-like effects, which may suppress the accumulation of plant defense compound, such as phytoalexin, upon infection by the generalist fungal pathogen *Neofusicoccum parvum* in grapevine [72]; (iv) asparagine, which O157:H7 can convert into aspartate [73], thereby feeding multiple metabolic pathways for growth; (v) D-(-)-quinic acid, an organic acid that may dampen plant defenses by modulating signaling pathway of stress-associated hormones such jasmonic acid [74].

To reduce the ability of O157:H7 to colonize the lettuce apoplast environment, the inhibitory cocktail contained: (i) trehalose, an osmoprotectant sugar that enhances plant growth and tolerance to biotic stressors [75, 76], although, trehalose might have the same effect on bacteria [77, 78]; (ii) glycerol, an osmoprotectant and carbon source for O157:H7 [79, 80], at high concentrations, however, it can inhibit microbial growth via osmotic or metabolic stress [81, 82]; iii-iv) benzoic acid and lactic acid, both well-characterized organic acids with antimicrobial propertied against O157:H7 [83–86]; v) alanine, an amino acid unknown to have an effect on bacterial survival. However, alanine and its derivatives were identified as DAMs and it was included in this cocktail to maintain its chemical balance across all cocktails.

To create a leaf apoplastic environment potentially conducive to O157:H7 growth, the promoting cocktail contained: (i) glucose, providing a carbon source for the bacterium; (ii) maltose, a disaccharide that supports O157:H7 colonization in diverse environments, including the murine intestine [87], bovine digestive contents [73], and lettuce leaf lysates [88]; (iii) succinic acid, an organic acid intermediate of the tricarboxylic acid (TCA) cycle may serve as a carbon source for *E. coli* growth in culture [89]. However, at high levels this compound, like other organic acids, may be toxic for this bacterium due to extremely reduction of the environmental pH [90, 91]; (iv) α-ketoglutaric acid, an organic acid intermediate of the TCA cycle, a pathway enriched in O157:H7 transcriptomes after lettuce leaf inoculation [36] and linked to bacterial fitness during urinary tract infection [92]; (v) glycine, an non-essential amino acid that O157:H7 produces from lettuce-derived choline for osmoprotection in injured leaves at pre-harvest and cut lettuce at post-harvest [93]. Glycine protects O157:H7 against acidic environments, such as the leaf apoplast, by altering its lipid membrane composition [80].

Overall, the modified chemical environments of lettuce leaves were associated with shifts in O157:H7 population dynamics as predicted (Fig. 5), indicating that manipulation of metabolite profiles can significantly influence pathogen persistence under the conditions tested. Although the inoculation approaches used here are widely employed in phyllosphere studies, they may not fully reflect natural contamination routes or deposition patterns and could influence bacterial colonization dynamics. A full mechanistic interpretation, however, is constrained by the complex chemistries of the pre-existing and inducible metabolic changes in these leaf niches, as well as the potential non-specific osmotic and pH-related effects of multi-compound cocktails on bacterial growth. For instance, a single metabolite could exert a dominant effect that masks the contribution of others, while interactions or synergistic effects among metabolites may also contribute to the O157:H7 population outcome. Moreover, the specific roles of these metabolites in the plant–bacterium system remain uncertain, as individual compounds may act on either organism, or on both, through distinct processes, adding complexity to causal inference. Future studies specifically designed to isolate individual metabolites, and their interactions will be required to disentangle these effects. Nonetheless, this study provides the first steps toward identifying candidate chemical features associated with bacterial population dynamics.

Noteworthily, we validated the effects of a few selected metabolite cocktails, however, the extensive metabolomic datasets (Datasets 5–6) generated in this study are valuable resources to breeders and geneticists to explore additional candidates influencing O157:H7 persistence in the phyllosphere. In particular, secondary metabolites such as polyphenols, which were not included in the functional validation assays, may exert antimicrobial effects [19, 34] or act as significant signaling compounds during plant-microbe interactions [94]. Targeted investigation of these compounds in future studies could further illuminate how lettuce chemical diversity shapes pathogen survival and guide plant-based strategies for food safety interventions.

## Conclusion

Exometabolomic profiling of multiple lettuce genotypes revealed that the chemical environment of the phylloplane and leaf apoplast contributes to the differential persistence of O157:H7 among genotypes. Further analyses of the profiles allowed the identification of compound signatures associated with either bacterial decline, persistence, or growth highlighting the contrasting influence of osmolytes, organic acids, and amino acids on pathogen survival. The influence of exometabolomic profiles on shifts in bacterial population sizes *in planta* was experimentally validated, demonstrating that metabolite supplementation can reproducibly suppress or promote O157:H7 survival on and within leaves, supporting a causal link between plant metabolite composition and bacterial fitness. These findings offer insight into metabolic traits that influence microbial survival useful to guide strategies for breeding or managing crops with reduced capacity to harbor enteric pathogens.

## Methods

### Lettuce genotypes and growth conditions

A set of 31 cultivated lettuces (*Lactuca sativa* L.), representing seven different horticultural types, were included in this study. Seeds from lettuce accessions and cultivars were obtained from seed repositories located at the University of California, Davis and the United States Department of Agriculture, Agricultural Research Service, Salinas, CA. All experimental research on these cultivated plants was conducted in compliance with relevant institutional and national guidelines. Genotypes received a laboratory code to not disclose sensitive information. Plants were grown in 12-liter pots (one plant per pot) containing a commercial growing mix (Sun Gro^®^ Sunshine^®^ #1 Grower Mix with RESiLIENCE™, Agawam MA, USA) at 18 ± 1 °C, 75 ± 4% relative air humidity, and photosynthetically active light intensity of 240 ± 10 µmol/m^2^/s with a 12-h photoperiod. Seven genotypes were maintained in the controlled environment room, while the remaining 24 genotypes were transported to Salinas, CA. These plants were grown under outdoor environmental conditions (Fig. S8) identical to commercial lettuce production in the main U.S. lettuce-producing area. After four weeks, these plants were transported to Davis, CA, acclimatized to the growing conditions described above for four days, and then used for subsequent studies.

### Preparation of O157:H7 inoculum

O157:H7 strain 86 − 24 (National Center for Biotechnology Information accession number PRJNA1073941) was streaked from a frozen glycerol stock on Low Salt Luria-Bertani (LSLB) agar medium (10 g/L tryptone, 5 g/L yeast extract, 5 g/L NaCl, 15 g/L agar) supplemented with 50 µg/mL streptomycin (Thermo Fisher Scientific, Waltham, MA, USA) and incubated overnight at 28 ^⸰^C. A single colony from these agar plates was inoculated into liquid LSLB broth and cultures were kept in an orbital shaker incubator set at 200 rpm and 28 °C until reaching an OD_600_ 0.85-1. Cultures were centrifuged at 5000 x*g* at 4 °C for 5 min and the cell pellet was resuspended with sterile deionized water (SDW).

### Plant inoculation and leaf sampling

The third fully expanded leaf of 4-week-old plants was inoculated with either 1 × 10^6^ CFU/mL O157:H7 inoculum or SDW as a mock control (Fig. S3). Inoculation of the leaf apoplast was performed using a needleless syringe following the protocol described by Katagiri et al. (2002). In addition to apoplastic infiltration, the surface of each leaf was inoculated by dipping into 200 mL of inoculum or mock solution containing 0.03% Silwet L-77 (Lehle Seeds Co., Round Rock, TX, USA) for 5 s. Plants were dried for 3 h and returned to the growth chamber. At 1- and 7-days post inoculation (DPI), leaves were harvested and placed in a sterile petri dish with their excision wound covered with parafilm. Images of each leaf were taken on a gridded background with a scale to estimate the surface area using ImageJ [95]. Three different plants (*n* = 3) were sampled per treatment at each time point.

### Exudate sampling and bacterial enumeration

Sampled leaves were individually rinsed with 10 mL of sterile phosphate buffered saline (PBS; 137 mM NaCl, 2.7 mM KCl, 4.3 mM Na_2_HPO_4_; 1.47 mM KH_2_PO_4_) by shaking at 125 rpm for 10 min (Fig. S3). The surface wash fluid (SWF) was obtained by collecting the rinse of each leaf. Water soluble metabolites and O157:H7 within the same sample were recovered by infiltrating the leaves with PBS, carefully blotted dry, and centrifuged at 500 x*g* for 8 min at 4 ^⸰^C to collect the apoplastic wash fluid (AWF) [32, 96]. This extraction method has been previously validated and shown to result in only marginal cytoplasmic contamination, as assessed by the activity of the cytoplasmic enzyme glucose‑6‑phosphate dehydrogenase [32].

An aliquot of SWF and AWF were used to enumerate O157:H7 population via serial dilution plating on LSLB, following published procedure [97]. The remaining exudate samples were filter-sterilized with a 0.22 μm filter, frozen on ice, and stored at -80 ^⸰^C.

Recovered bacterium population size from the leaf surface or apoplast was estimated as colony forming unit (CFU)/cm^2^ of leaf surface or CFU/mL AWF. Significant differences among bacterial net growth means were determined using ANOVA coupled with the Scott-Knott post-hoc test (α = 0.05), both performed in R [98].

To estimate the relationship between the bacterial net growth on the leaf surface (epiphytes) and in the leaf apoplast (endophytes) across lettuce genotypes, Pearson´s correlation analysis was performed using the cor.test function in R.

### Growth of O157:H7 in AWF cultures

Apoplastic wash fluids were recovered via the infiltration-centrifugation procedure described above. AWF with an initial 2.5 × 10^4^ O157:H7 CFU/mL concentration were added to a 96-well plate and incubated overnight in a BioTek Synergy HTX plate reader (Agilent Technologies, Folsom, CA, USA) at 30 ^⸰^C with constant orbital-shaking (250 rpm). Readings at optical density 600 nm (OD_600_) were measured every 30 min for 18 h. Each biological replicate consisted of the combined fluid extracted from three leaves of an individual plant, and three biological replicates were used (*n* = 3). The negative and positive controls consisted of 1x solution of M9 salts (MilliporeSigma, Burlington, MA, USA) and LSLB, respectively.

The maximum growth rate (µmax) and the maximum population density (carrying capacity) were estimated using the growthrates package in R [99]. µmax was calculated by fitting a linear model to the natural Log-transformed exponential phase of each growth curve. Significant differences among group means (*n* = 3) were assessed via ANOVA followed by a Scott-Knott post-hoc test (α = 0.05) in R. Pearson’s correlation analysis, performed using the cor.test function in R, was used to assess the relationship between µmax or carrying capacity and apoplastic bacterial population size at 1 or 7 DPI.

### Metabolome data collection

Based on the phenotypic variability of O157:H7 population net growth, 14 lettuce genotypes were selected for metabolomic profiling of their leaf surface and apoplastic exudates (Fig. S3). Samples were submitted to the University of California, Davis, West Coast Metabolomic Center (https://metabolomics.ucdavis.edu) for metabolomic profiling of primary metabolites and polyphenols and flavonoids. Samples were randomized during extraction and mass spectrometry analysis to minimize potential batch- and run‑order effects.

Primary metabolites were detected via gas chromatography coupled to time-of-flight mass spectrometry (GC-TOF MS) utilizing a Rtx-5Sil MS column (Restek Corporation, Bellefonte, PA, USA) coupled with a Pegasus IV mass spectrometer (LECO Corporation, St. Joseph, Michigan, USA). All data acquisition by GC-TOF MS analysis and quality control followed the chromatography parameters described by Fiehn et al. (2008).

Polyphenols and flavonoids were quantified by UHPLC–MS/MS (Vanquish UHPLC coupled to Q-Exactive HF Orbitrap; Thermo Fisher) using an Acquity Premier BEH C18 column (1.7 μm, 2.1 × 50 mm; Waters). Samples were extracted in 80:20 MeOH: H₂O, dried, and reconstituted in 75:25 H₂O: ACN with internal standards. After vortexing, sonication, and centrifugation, supernatants were injected (0.1–5 µL). Separation used a 1–99% ACN + 0.1% formic acid gradient (0.6 mL/min). MS data were acquired in positive and negative ESI modes (m/z 80–1200) at a 60,000 resolution for MS and 15,000 for dd-MS/MS, with 20–40% normalized collision energy. The instrument was tuned and calculated according to manufacturer’s guidelines.

Metabolomic data were deposited in the EMBL-EBI database (MetaboLights: https://www.ebi.ac.uk/metabolights/index) under the accession number REQ20250721211973.

### Metabolome data analysis

Metabolomic data were grouped into four datasets: primary metabolites at 1 DPI (Dataset S1), polyphenols and flavonoids at 1 DPI (Dataset S2), primary metabolites at 7 DPI (Dataset S3), and polyphenols and flavonoids at 7 DPI (Dataset S4). For each dataset, metabolite peak heights were normalized by the average peak-sum of each metabolite (mTIC_average_) [48], followed by Log₁₀ transformation and autoscaling (unit variance standardization) using the freeware MetaboAnalyst 6.0 (https://www.metaboanalyst.ca).

Relationships among the variables biological replicate, lettuce genotype, and inoculation type were explored using principal component analysis (PCA). PCA coordinates were computed in MetaboAnalyst 6.0. To further investigate patterns of metabolite profiles across samples, hierarchical clustering analysis was conducted with all identified metabolites through each platform. Clustering analysis was performed using the average of normalized metabolite values in the pheatmap package in R, using Euclidean distance for both rows and columns, and Ward’s minimum variance method (Ward.D2) for linkage.

### Identification of differentially accumulated metabolites

Differentially accumulated metabolites (DAMs) were determined by comparing normalized metabolite peak values from mock and O157:H7 treatments for each genotype using MetaboAnalyst 6.0. DAMs were considered significant when the Student’s *t*-test showed a *p* < 0.05 and an absolute Log₂ fold change (FC) ≥ 1 when comparing O157:H7- versus mock-treated samples. Differential metabolite analysis was conducted within an exploratory framework using unadjusted p‑values, as multiple testing correction substantially reduced potential DAM discovery given the high dimensionality of the dataset and limited replication (*n* = 3). DAMs were interpreted conservatively and used to guide downstream validation. To identify shared and unique DAMs among lettuce genotypes, intersection analyses were performed using the UpSetR package in R.

### Pathway enrichment analysis

DAMs were used as input for pathway enrichment analyses in MetaboAnalyst 6.0. The hypergeometric test was used to evaluate statistical enrichment of metabolites within known KEGG pathways, with raw p-values < 0.05 considered significant. Pathway topology analysis was performed using relative-betweenness centrality to calculate a pathway impact score, which reflects the topological relevance of each mapped metabolite within a given pathway. Bubble plots were generated in R to visualize enriched pathways, with bubble size representing absolute Log_10_ p-value and color intensity indicating the pathway impact score.

### Experimental validation of metabolomic data

Comprehensive metabolome analyses enabled the design of cocktails of five metabolites to validate their influence on O157:H7 population net growth dynamics in lettuce. Cocktails contained 0.01 M for each of five metabolites in SDW, which were purchased from Thermo Fisher Scientific or MilliporeSigma (Burlington, MA, USA). The four different cocktails were named “promoting” or “inhibitory” according to their predicted effect on bacterial net growth on the leaf surface or in the leaf apoplast. Cocktails were used to supplement the O157:H7 inoculum (1 × 10^6^ CFU/mL). A water supplemented bacterial inoculum was used as a control. Six genotypes were selected based on their phenotypes for bacterial net growth on either the leaf surface (04, 15, 38, 39, 44, and 45) or in the apoplast (02, 15, 31, 39, 44, and 45). Plants growth, inoculation, and bacterial enumeration were carried out as described above. Significant differences among the means (*n* = 3 plants) across the treatments were measured via ANOVA and Tukey (α = 0.05) using R. The experiment was repeated twice with independent batches of plants.

## Supplementary Information

Supplementary Material 1: Fig. S1. Pearson’s correlation analysis between bacterial net growth on the leaf surface (epiphytes) and leaf apoplast (endophytes) across lettuce genotypes that were color coded to aid visualization on the plot.

Supplementary Material 2: Fig. S2. Pearson’s correlation analyses between µmax (A, C) or carrying capacity (B, D) vs. apoplastic bacterial population size at 1 DPI (A, B) or 7 DPI (C, D) as indicated in the axis titles. DPI = days post inoculation.

Supplementary Material 3: Fig. S3. Flow chart of the experimental procedure to collect leaf exudates for exometabolomic analysis to identify metabolites associated with bacterial growth or decline on the leaf surface and/or leaf apoplast. SWF = surface wash fluid, AWF = apoplast wash fluid. Diagram was created in Biorender.com.

Supplementary Material 4: Dataset S1. Primary metabolite abundance values measured via gas chromatography coupled with time-of-flight mass spectrometry (GC-TOFMS) from exudates in the apoplast (A) or on leaf surface (S) of E. coli O157:H7-inoculated (A_E, S_E) or mock-inoculated (A_M, S_M) plants at one day post inoculation (1 DPI). The numbers on the column title indicate the lettuce genotype code. Results are shown as normalized values (sum normalized, Log10 transformed and auto-scaled) calculated with MetaboAnalyst 6.0 (https://www.metaboanalyst.ca/) for three biological replicates (BR) and their average. Normalization was performed across treatments within sample type (leaf surface and apoplast). Metabolite identifications were annotated according to the PubChem database utilizing the OMU R package.

Supplementary Material 5: Dataset S2. Polyphenol and flavonoid metabolite abundance values measured via exactive tandem mass spectrometry (MS/MS) from exudates in the apoplast (A) or on leaf surface (S) of E. coli O157:H7-inoculated (A_E, S_E) or mock-inoculated (A_M, S_M) plants at one day post inoculation (1 DPI). The numbers on the column title indicate the lettuce genotype code. Results are shown as normalized values (sum normalized, Log10 transformed, and auto-scaled) calculated with MetaboAnalyst 6.0 (https://www.metaboanalyst.ca/) for three biological replicates (BR) and their average. Normalization was performed across treatments within sample type (leaf surface and apoplast). Metabolite identifications were annotated according to the PubChem database utilizing the OMU R package.

Supplementary Material 6: Dataset S3. Primary metabolite abundance values measured via gas chromatography coupled with time-of-flight mass spectrometry (GC-TOFMS) from exudates in the apoplast (A) or on leaf surface (S) of E. coli O157:H7-inoculated (A_E, S_E) or mock-inoculated (A_M, S_M) plants at seven days post inoculation (7 DPI). The numbers on the column title indicate the lettuce genotype code. Results are shown as normalized values (sum normalized, Log10 transformed and auto-scaled) calculated with MetaboAnalyst 6.0 (https://www.metaboanalyst.ca/) for three biological replicates (BR) and their average. Normalization was performed across treatments within sample type (leaf surface and apoplast). Metabolite identifications were annotated according to the PubChem database utilizing the OMU R package.

Supplementary Material 7: Dataset S4. Polyphenol and flavonoid metabolite abundance values measured via exactive tandem mass spectrometry (MS/MS) from exudates in the apoplast (A) or on leaf surface (S) of E. coli O157:H7-inoculated (A_E, S_E) or mock-inoculated (A_M, S_M) plants at seven days post inoculation (7 DPI). The numbers on the column title indicate the lettuce genotype code. Results are shown as normalized values (sum normalized, Log10 transformed, and auto-scaled) calculated with MetaboAnalyst 6.0 (https://www.metaboanalyst.ca/) for three biological replicates (BR) and their average. Normalization was performed across treatments within sample type (leaf surface and apoplast). Metabolite identifications were annotated according to the PubChem database utilizing the OMU R package.

Supplementary Material 8: Fig. S4. Relationship among samples considering all detected primary (A and C) or polyphenols (B and D) metabolites in the surface wash fluid (SWF) or apoplastic wash fluid (AWF) collected from the lettuce genotypes at 1 day post inoculation (DPI) (A and B) and at 7 DPI (C and D). Mock- and O157:H7-treated samples from each genotype (vertical strips) are represented by empty and filled squares, respectively. Principal component analysis was conducted with MetaboAnalyst 6.0 software using normalized peak heights (Log10 transformation and autoscaling functions) across all genotypes. Plots were created with individual genotypes to aid visualization

Supplementary Material 9: Fig. S5. Hierarchical clustering of all metabolites identified with each metabolomic platforms, primary metabolites (PM) (A, B, E, F) and polyphenols and flavonoids (PF) (C, D, G, H), at one (A-D) or seven (E-H) days post inoculation. Surface wash fluid (SWF) samples (A, C, E, G) and apoplast wash fluid (AWF) samples (B, D, F, H) were analyzed separately. Clustering analysis was performed with the average of normalized metabolite values (Datasets S1-4) using the pheatmap package in R, according to a Euclidean distance for both rows and columns and Ward’s minimum variance method (Ward.D2) for linkage. Genotype codes and inoculation type (mock or O157:H7) are color coded and depicted at the top of the cluster lanes.

Supplementary Material 10: Dataset S5. Comparisons between metabolite accumulation in O157:H7- vs. mock-inoculated samples (surface or apoplast wash fluids) for the indicated genotype collected at one day post inoculation (1 DPI). Results are expressed as fold change (FC) and Log2 FC, and statistical significance between the means was calculated with unpaired Student´s t-test in the MetaboAnalyst 6.0 software. Differentially accumulated metabolites (DAMs) were identified based on *p* < 0.05. Cells labeled with NA correspond to missing values for that metabolite in each sample. t-stat = t-statistic (statistic associated with Student’s t-test). FDR = false discovery rate. Metabolites selected for the promoting or inhibitory cocktail and their derivatives are highlighted in green or red, respectively.

Supplementary Material 11: Dataset S6. Comparisons between metabolite accumulation in O157:H7- vs. mock-inoculated samples (surface or apoplast wash fluids) for the indicated genotype collected at seven days post inoculation (7 DPI). Results are expressed as fold change (FC) and Log2 FC, and statistical significance between the means was calculated with unpaired Student´s t-test in the MetaboAnalyst 6.0 software. Differentially accumulated metabolites (DAMs) were identified based on *p* < 0.05. Cells labeled with NA correspond to missing values for that metabolite in each sample. t-stat = t-stat = t-statistic (statistic associated with Student’s t-test). FDR = false discovery rate. Metabolites selected for the promoting or inhibitory cocktail their derivatives are highlighted in green or red, respectively.

Supplementary Material 12: Dataset S7. Pathway enrichment analysis of metabolites significantly differentially accumulated (bacterium vs. mock) in the surface wash fluid (SWF) and apoplast wash fluid (AWF) of the lettuce genotypes. Significantly enriched pathways (*p* < 0.05) were identified using KEGG-based pathway analysis in MetaboAnalyst6.0 via the hypergeometric test.

Supplementary Material 13: Fig. S6 Intersection analysis of significantly (*p* < 0.05) differentially accumulated metabolites (DAMs) detected from the comparison between O157:H7- and mock-inoculated samples of each lettuce genotype. Shared and unique accumulated metabolites (O157:H7 vs. mock) detected in the surface wash fluid (SWF) (A) and apoplast wash fluid (AWF) (B) among lettuce genotypes were identified with the UpSetR package in R. The identity of these metabolites is presented in Dataset S8.

Supplementary Material 14: Dataset S8. Intersection analyses of significantly differentially accumulated metabolites (DAMs) detected in the surface wash fluid (SWF) and apoplast wash fluid (AWF) of each genotype were performed using the UpSetR package in R. Shared and unique metabolites hypo- and hyper-accumulated metabolites among lettuce genotypes are shown. Metabolites selected for the promoting or inhibitory cocktail are highlighted in green or red, respectively.

Supplementary Material 15: Fig. S7. Relative accumulation of selected metabolites and their derivates across lettuce genotypes. These metabolites were combined in cocktails based on their predicted inhibitory or promoting effect on O157:H7 growth. The heatmaps, generated with the pheatmap package in R, show the Log2 fold change (O157:H7- versus mock-inoculated plants) of metabolites used to supplement inoculations of the leaf surface (A) or apoplast (B). The values and statistical analysis are listed in Datasets S5 and S6.

Supplementary Material 16: Fig. S8. Maximum, minimum, and average temperatures recorded in Salinas, CA during the two field trials in 2021. Temperature data were obtained from the Monterey Peninsula Airport Station (https://forecast.weather.gov/data/obhistory/KMRY.html).

## Data Availability

Metabolomic data were deposited in the EMBL-EBI database (MetaboLights: https://www.ebi.ac.uk/metabolights/index) under the accession number REQ20250721211973. All additional data generated or analyzed during this study are included in this published article and its supplementary information files.
