# Supplementary figures and images for: Exometabolomic-enabled discovery of compounds associated with Escherichia coli O157:H7 population dynamics in the lettuce phyllosphere
Source: BMC Plant Biol. 2026 May 9;26:1114. doi: 10.1186/s12870-026-08917-9 (PMC13326550; doi:10.1186/s12870-026-08917-9)

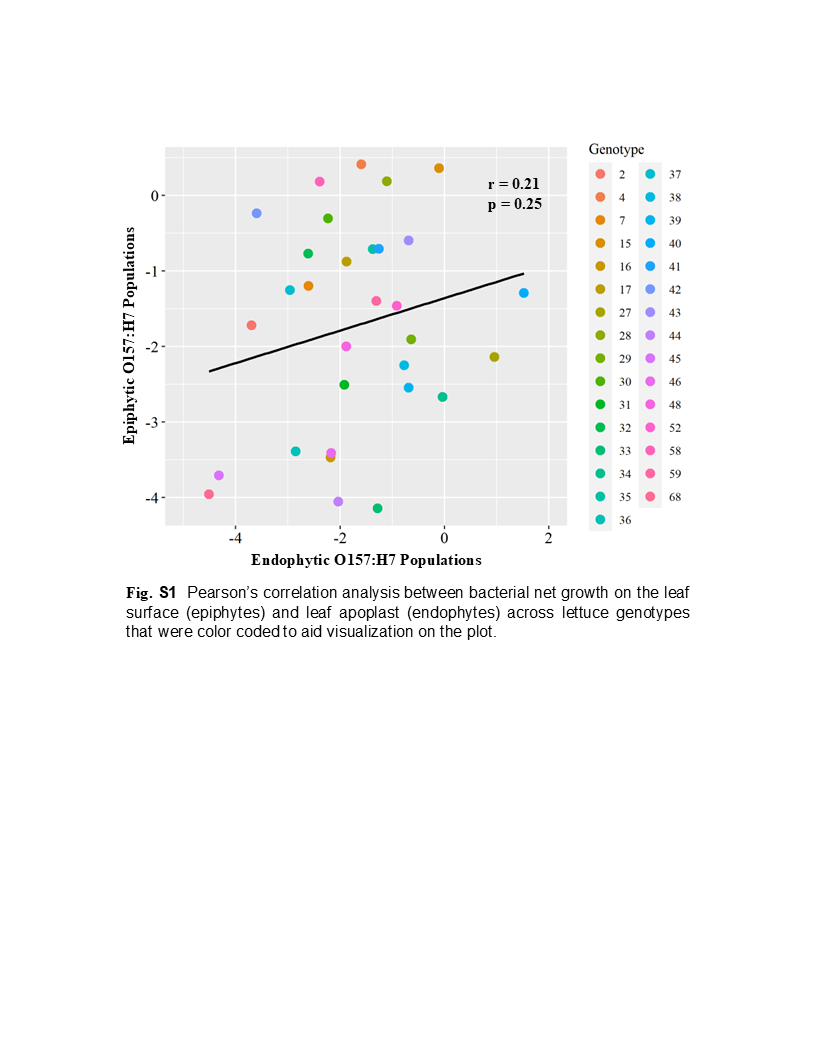

Supplement: Supplementary file 1 — Supplementary Material 1: Fig. S1. Pearson’s correlation analysis between bacterial net growth on the leaf surface (epiphytes) and leaf apoplast (endophytes) across lettuce genotypes that were color coded to aid visualization on the plot. [file 12870_2026_8917_MOESM1_ESM.tif]

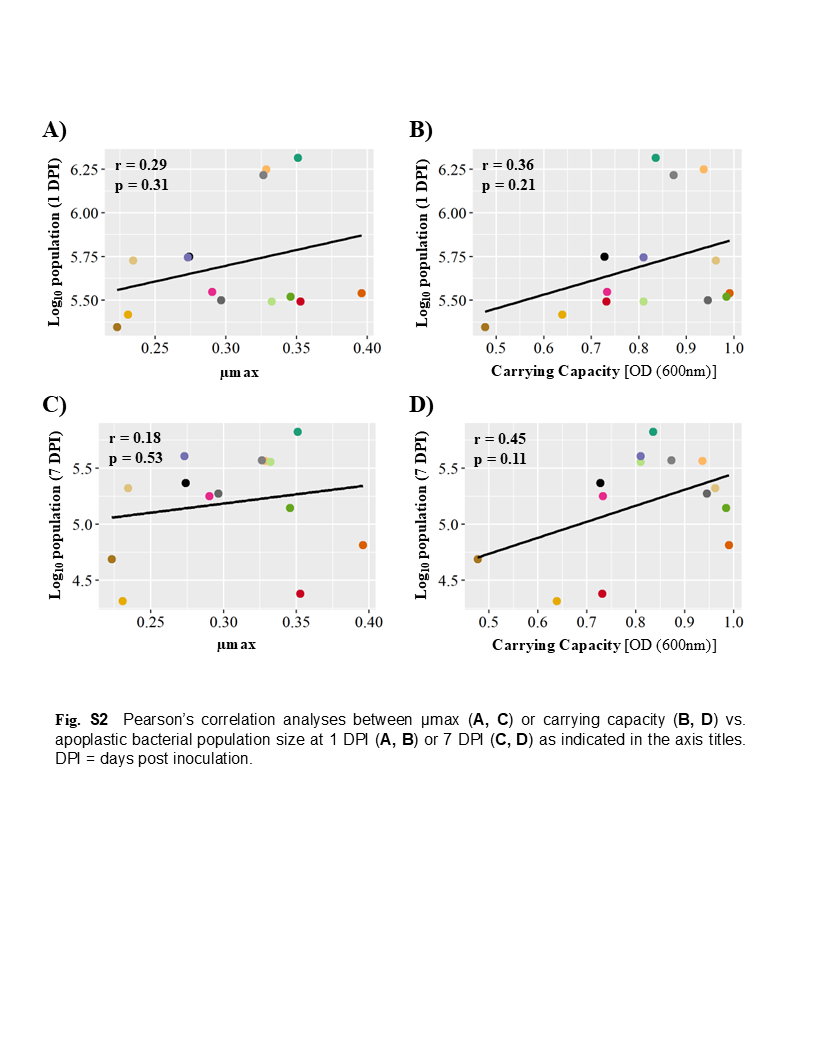

Supplement: Supplementary file 2 — Supplementary Material 2: Fig. S2. Pearson’s correlation analyses between µmax (A, C) or carrying capacity (B, D) vs. apoplastic bacterial population size at 1 DPI (A, B) or 7 DPI (C, D) as indicated in the axis titles. DPI = days post inoculation. [file 12870_2026_8917_MOESM2_ESM.tif]

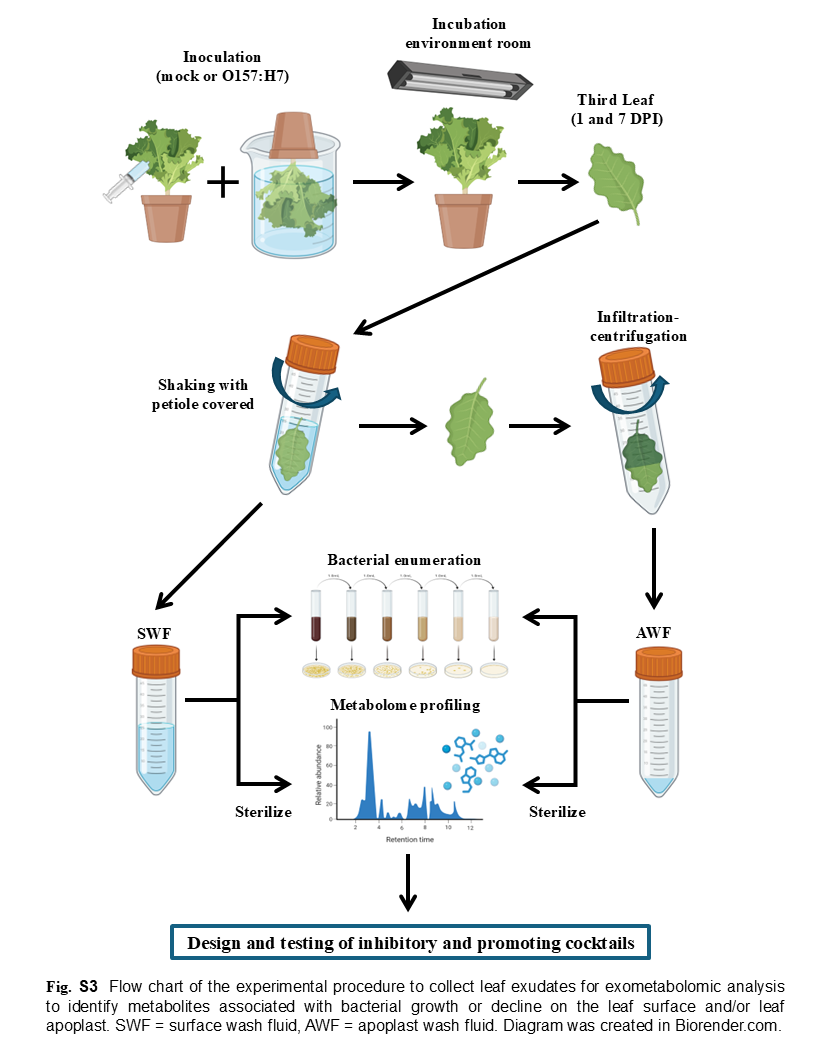

Supplement: Supplementary file 3 — Supplementary Material 3: Fig. S3. Flow chart of the experimental procedure to collect leaf exudates for exometabolomic analysis to identify metabolites associated with bacterial growth or decline on the leaf surface and/or leaf apoplast. SWF = surface wash fluid, AWF = apoplast wash fluid. Diagram was created in Biorender.com. [file 12870_2026_8917_MOESM3_ESM.tif]

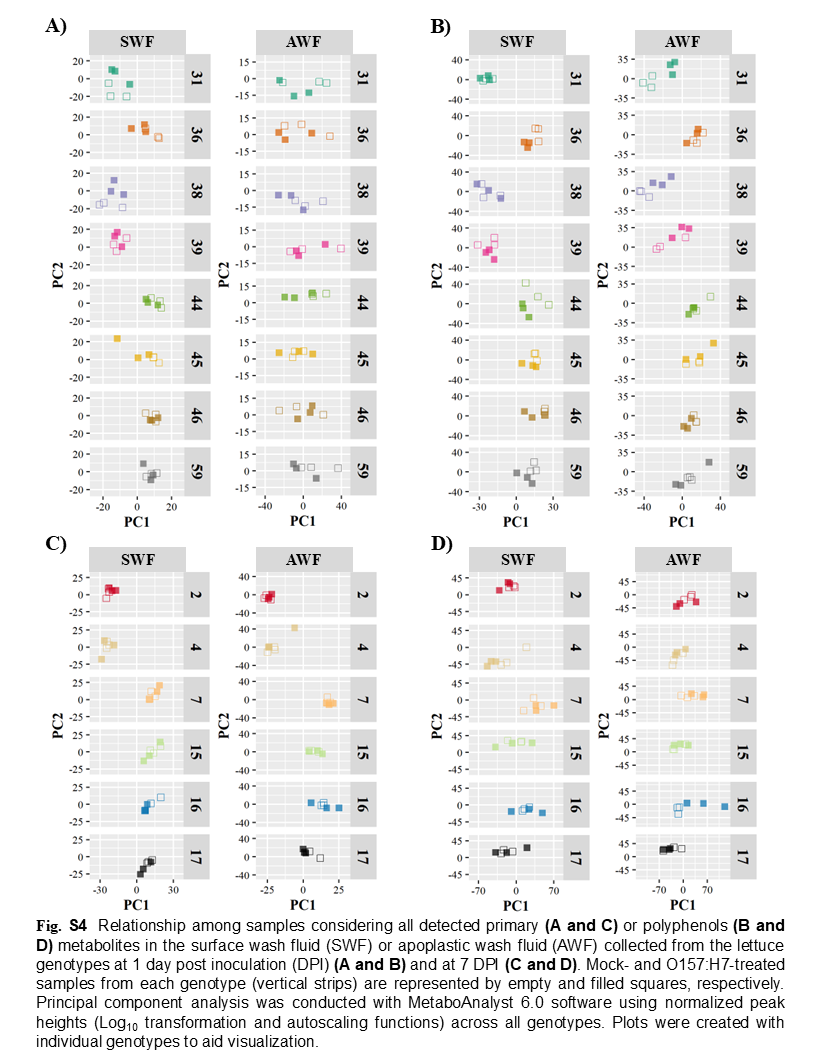

Supplement: Supplementary file 8 — Supplementary Material 8: Fig. S4. Relationship among samples considering all detected primary (A and C) or polyphenols (B and D) metabolites in the surface wash fluid (SWF) or apoplastic wash fluid (AWF) collected from the lettuce genotypes at 1 day post inoculation (DPI) (A and B) and at 7 DPI (C and D). Mock- and O157:H7-treated samples from each genotype (vertical strips) are represented by empty and filled squares, respectively. Principal component analysis was conducted with MetaboAnalyst 6.0 software using normalized peak heights (Log10 transformation and autoscaling functions) across all genotypes. Plots were created with individual genotypes to aid visualization [file 12870_2026_8917_MOESM8_ESM.tif]

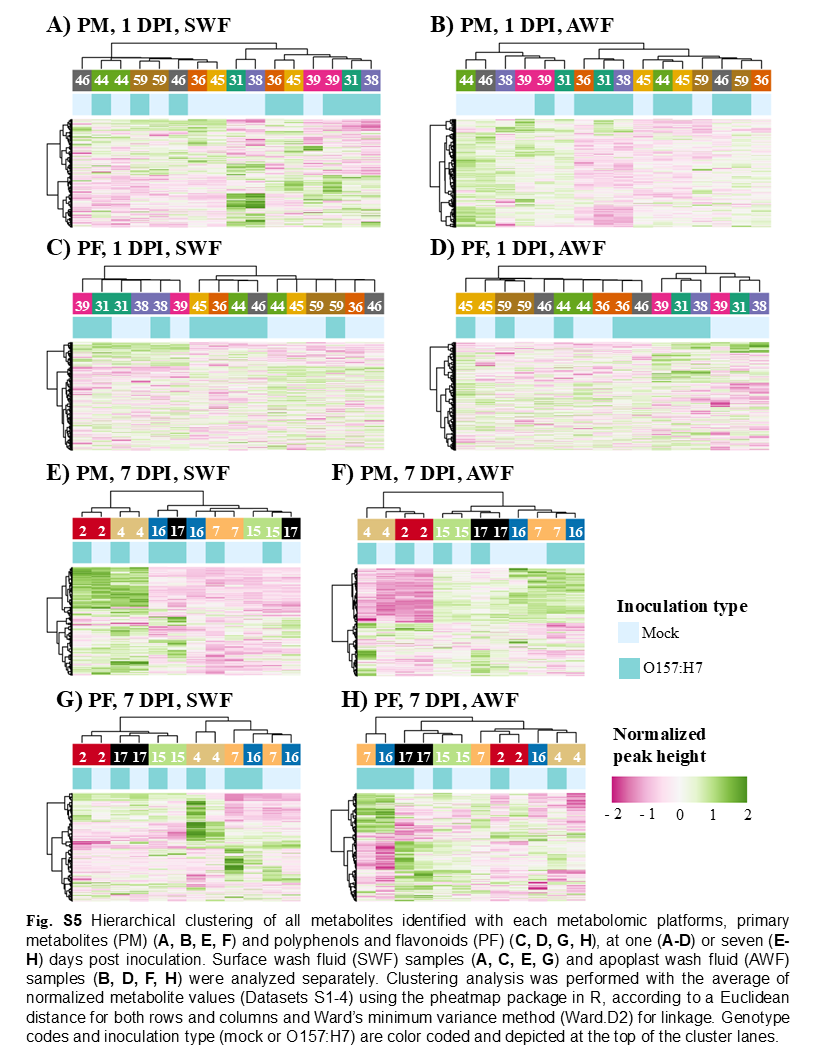

Supplement: Supplementary file 9 — Supplementary Material 9: Fig. S5. Hierarchical clustering of all metabolites identified with each metabolomic platforms, primary metabolites (PM) (A, B, E, F) and polyphenols and flavonoids (PF) (C, D, G, H), at one (A-D) or seven (E-H) days post inoculation. Surface wash fluid (SWF) samples (A, C, E, G) and apoplast wash fluid (AWF) samples (B, D, F, H) were analyzed separately. Clustering analysis was performed with the average of normalized metabolite values (Datasets S1-4) using the pheatmap package in R, according to a Euclidean distance for both rows and columns and Ward’s minimum variance method (Ward.D2) for linkage. Genotype codes and inoculation type (mock or O157:H7) are color coded and depicted at the top of the cluster lanes. [file 12870_2026_8917_MOESM9_ESM.tif]

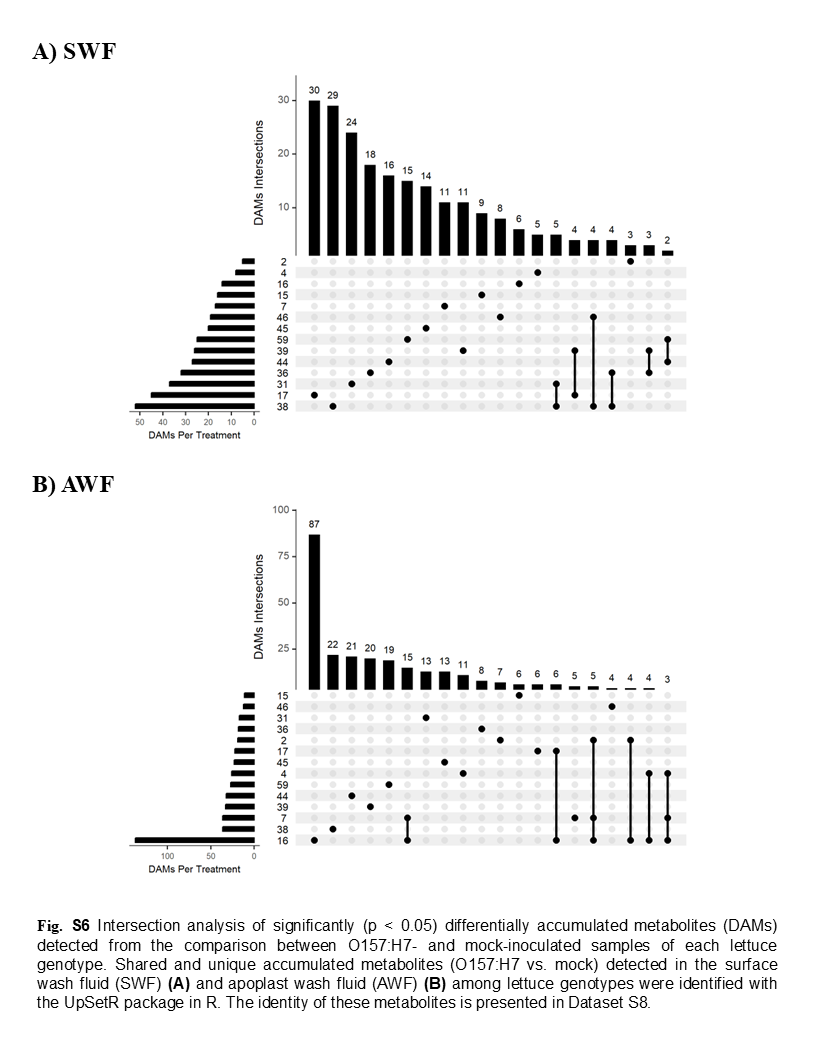

Supplement: Supplementary file 13 — Supplementary Material 13: Fig. S6 Intersection analysis of significantly (p < 0.05) differentially accumulated metabolites (DAMs) detected from the comparison between O157:H7- and mock-inoculated samples of each lettuce genotype. Shared and unique accumulated metabolites (O157:H7 vs. mock) detected in the surface wash fluid (SWF) (A) and apoplast wash fluid (AWF) (B) among lettuce genotypes were identified with the UpSetR package in R. The identity of these metabolites is presented in Dataset S8. [file 12870_2026_8917_MOESM13_ESM.tif]

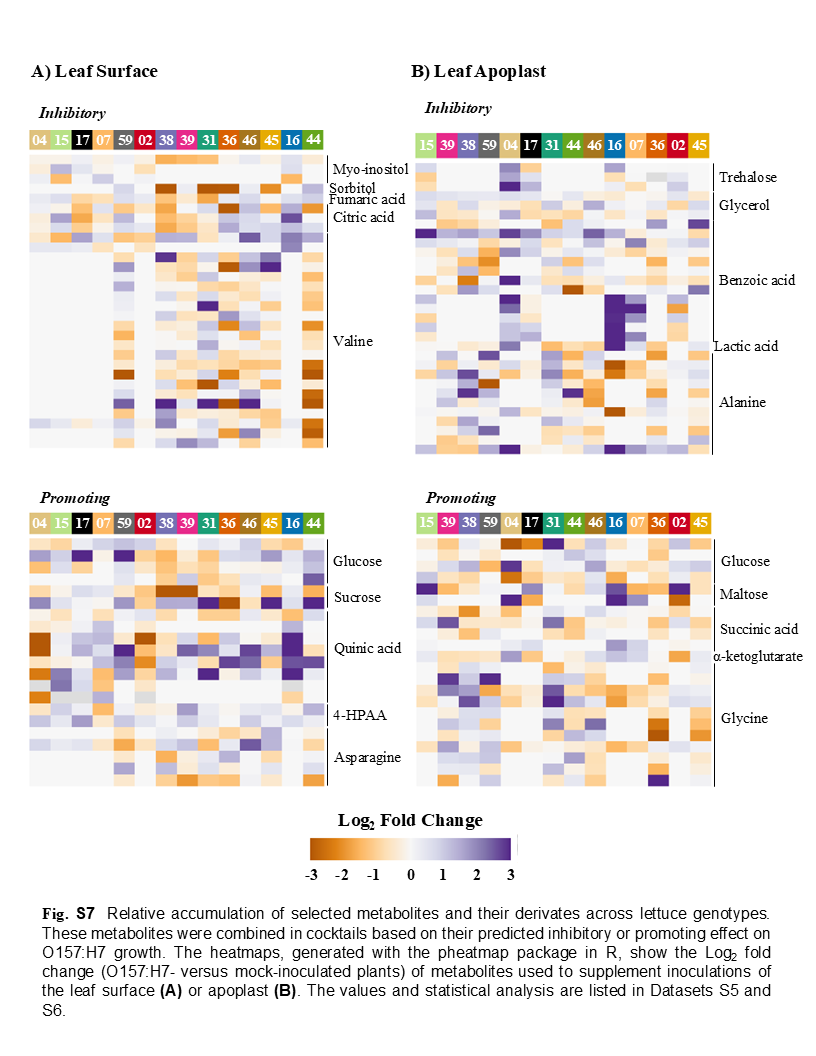

Supplement: Supplementary file 15 — Supplementary Material 15: Fig. S7. Relative accumulation of selected metabolites and their derivates across lettuce genotypes. These metabolites were combined in cocktails based on their predicted inhibitory or promoting effect on O157:H7 growth. The heatmaps, generated with the pheatmap package in R, show the Log2 fold change (O157:H7- versus mock-inoculated plants) of metabolites used to supplement inoculations of the leaf surface (A) or apoplast (B). The values and statistical analysis are listed in Datasets S5 and S6. [file 12870_2026_8917_MOESM15_ESM.tif]

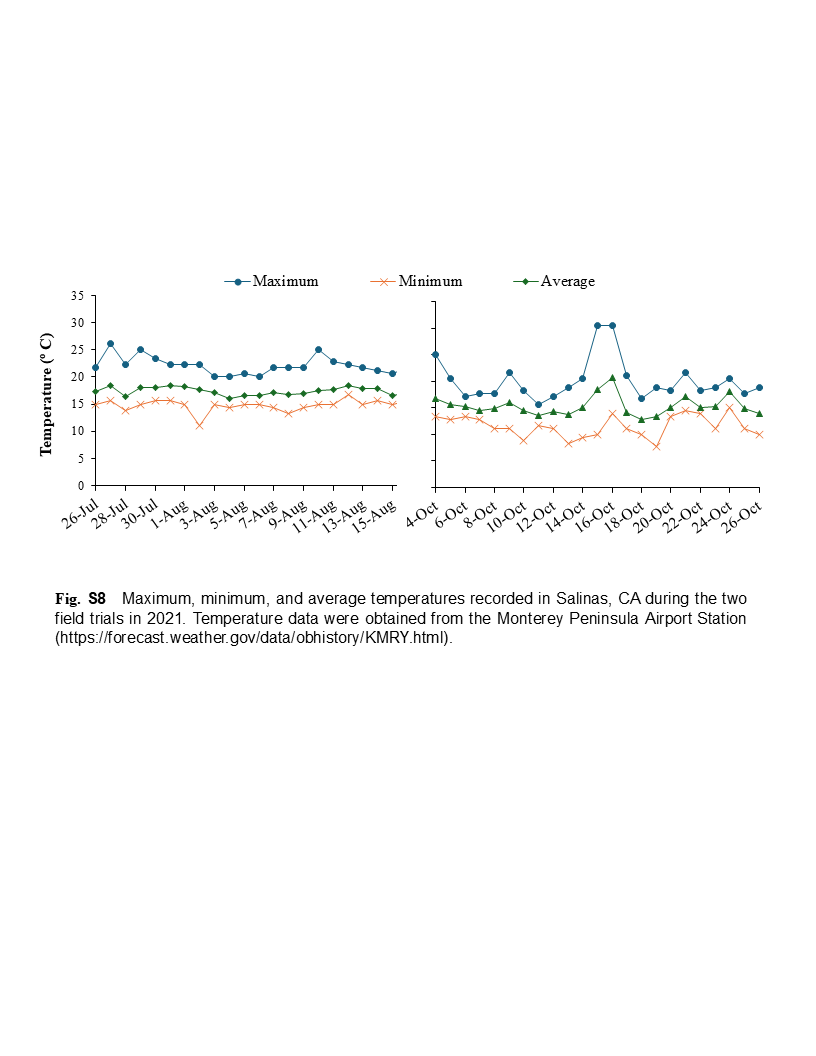

Supplement: Supplementary file 16 — Supplementary Material 16: Fig. S8. Maximum, minimum, and average temperatures recorded in Salinas, CA during the two field trials in 2021. Temperature data were obtained from the Monterey Peninsula Airport Station (https://forecast.weather.gov/data/obhistory/KMRY.html). [file 12870_2026_8917_MOESM16_ESM.tif]
